# Parental bonding styles in schizophrenia, depressive and bipolar patients: a comparative study

**DOI:** 10.1186/s12888-021-03177-3

**Published:** 2021-03-26

**Authors:** Aidin Abbaspour, Masoud Bahreini, Sherafat Akaberian, Kamran Mirzaei

**Affiliations:** 1 Nursing and Midwifery School, Bushehr University of Medical Sciences, Bushehr, Iran; 2 Nursing and Midwifery School, Shiraz University of Medical Sciences, Shiraz, Iran; 3 Community Medicine, Medical School, Bushehr University of Medical Sciences, Bushehr, Iran

**Keywords:** Bipolar disorder, Depression, Parental bonding, Schizophrenia

## Abstract

**Background:**

Numerous bio-psychosocial factors play a role in the etiology of psychiatric disorders. In this regard, the relationship between parents and their children is significantly involved in developing the offspring mental health. However, there is no clear-cut answer as to which parental bonding style is more strongly associated with psychiatric diseases of patients. This study aimed to compare parental bonding styles in patients with schizophrenia, depression, and bipolar disorder in Bushehr province, Iran in 2018.

**Methods:**

In this cross-sectional comparative study, 130 patients with schizophrenia, depression, and bipolar disorder who referred to four outpatients psychiatric centers in Bushehr were selected using quota sampling. The patients were assessed and compared in terms of parental bonding styles. Data were collected using a valid and reliable parental bonding instrument (PBI). Data were analyzed using SPSS software (ver. 22), Chi-square and Kruskal-Wallis tests at a significant level of 0.05.

**Results:**

Results showed that the optimal parental bonding style (low control, high care) in bipolar disorder (43.05%), major depression (47.7%), and schizophrenia (38.5%) was the most prevalent style of parental bonding; however, 62.30% of the above patients suffered from inefficient paternal bonding styles and 51.53% from inefficient maternal bonding styles. Furthermore, the patients’ maternal bonding styles were significantly different (*p* = 0.007) while their paternal bonding styles did not show any significant differences (*p* = 0.848).

**Conclusions:**

Most of the patients with psychiatric disorders were affected by ineffective parenting styles. The results also confirmed that despite the several bio-psycho-social factors involved in the development of psychiatric disorders, the crucial roles of parents, especially mothers, should not be ignored. It was further suggested that parents and parental bonding were important and fundamental factors for mental health promotion.

**Supplementary Information:**

The online version contains supplementary material available at 10.1186/s12888-021-03177-3.

## Background

Psychiatric disorders are increasing and significantly affecting the public health all over the world [1]. These disorders usually occur with psychological and behavioral manifestations which lead to significant functional deficiencies. Among the psychiatric disorders, schizophrenia, bipolar disorder, and depression are particularly important with the highest bed occupancy rates in psychiatric wards. Schizophrenia is a severe psychiatric disorder involving delusions and hallucinations. Depression is characterized by feeling sad, anhedonia, guilt, and suicidal thoughts, and bipolar is defined as periods of depression, elevated mood, over activity, reduced need for sleep, and pressured speech [2]. There are 20 million people with schizophrenia, about 45 million cases of bipolar disorder, and 264 million people with major depression around the world [3]. In Iran, demographic studies have reported the prevalence of these three disorders to be 25 to 31% [4].

Such diseases entail serious ramifications for patients and affect their interpersonal relationships and personal and social performance. Feelings of helplessness, hopelessness, worthlessness, stigma, fear, vulnerability, and low quality of life are among such consequences [3]. Various biological, familial, psychosocial, and spiritual factors are involved in the etiology of these disorders. These factors can be considered as predisposing, precipitating, and perpetuating factors [5]. Family, as one the most important factors, could impact the mental health of the members [6]. In Iran, the recent socio-economic changes, advances in technology, fading family traditions, more women’s social activities, and employment of both parents outside home have led to the investigation of the role of family, especially parents, in the development of children’s mental health.

Over the past decade, some studies have reported that the quality of life is lower in patients with mental disorders than the general public [7]. These investigations have raised questions led to further research on relationships among family members and its effects on children’s mental health. Accordingly, the available scientific evidence has hypothesized that family or parents can have critical parts in the occurrence of certain mental disorders. Bowlby’s concepts of attachment have been applied to psychopathology and to the psychological understanding of psychiatry disorders. Bowlby related deficient/pathological parenting with lack of care and excessive control/protection dimensions [8]. On this account, family relationships, particularly parents’ roles in the family and children’s mental development, can be considered and studied as strong variables influencing the children’s future mental health [9]. In this regard, the concept of parental bonding has been specifically taken into consideration by researchers worldwide [10]. According to Bowlby’s theory of attachment, parents who are unable to either establish warm, loving, and close relationships with their children or provide the necessary environment for their children’s independent development, raise their children in an atmosphere of anxiety, leading to psychological disorders [11]. The concept of parental bonding also focuses on the quality of the relationship between parents and offspring throughout their life. A warm relationship with adequate parental care and control (optimum parental bonding) plays an important role in the development children’s mental health. On the contrary, dysfunctional parenting leads to problems in interpersonal relationships and psychiatric disorders caused by the cold relationship between parents and children, inadequate care, and excessive control or rejection of children [12]. As a leading researcher in this field, Parker has introduced the dual styles of parental bonding in a continuum. The first style, “caring parental bonding”, reflects a warm, close, and empathetic relationship as opposed to a cold, rejecting, and neglecting relationship. The second style is the “over protection” or “control parental bonding” which involves parents’ severe control and protection over their children, leading to the non-completion of independence in the children [13].

Studies focusing on the concept of parental bonding have examined the association between parental bonding in childhood and psychiatric symptoms in adulthood [14, 15]. For instance, a study in Brazil compared the parental bonding styles of parents of schizophrenic and bipolar patients. Based on their results, there were significant differences between parental bonding styles in these two diseases, especially among the maternal bonding styles [16]. In a review article, researchers discussed research concerning the association between parental bonding in childhood and psychiatric symptoms in adulthood. They found that neglectful relationships (low care) and overprotection seem to represent risk factors for the development of psychiatric symptoms in offspring [17]. Another study was conducted to determine the relationship between parental bonding and attitudes toward suicide in medical students in Japan. The researchers concluded that high levels of maternal care ensured reduced suicidal ideation [18]. In Iran, results of a cross-sectional study on university students showed that the students who experienced a less caring parental bonding style, had significantly higher psychological symptoms, particularly depression, anxiety, and paranoia [19]. In this study, researchers investigated the healthy dormitory students of a university in Iran. On the contrary, the results of another study in Iran revealed that depression and lack of self-confidence rates in children significantly increased in parenting styles in which parents exaggerated in caring for or controlling their children [20].

Health care providers should target both patients and their families, necessitating an accurate evaluation of family performance [21]. A review of the related literature also indicates that relatively few studies have addressed psychiatric patients, and most have mainly focused on samples other than psychiatric patients. This research gap is more evident in Iran. Due to the increasing prevalence of psychiatric disorders and the undeniable role of family in the development of children’s mental health, there is an urgent need for more detailed studies in this field.

### Aim and hypotheses

This study aimed to evaluate and compare parental bonding styles in patients with schizophrenia, depression, and bipolar disorders in Bushehr, Iran. Based on the aforementioned studies [16 and 17] and the attachment theory, we have hypothesized that: (1) maternal and paternal care score in patients with schizophrenia would be higher than patients with BD and MDD (mood disorders), (2) maternal and paternal control score in patients with schizophrenia would be lower than patients with BD and MDD and (3) non optimal parental bonding styles would be more common in patients with schizophrenia than patients with BD and MDD.

## Methods

### Design and participants

In this cross-sectional comparative study, conducted in the adult psychiatric clinics in Bushehr, southwestern Iran, the study population consisted of Patients with confirmed diagnosis of schizophrenia, BD and MDD. Diagnostic interviews include psychiatric interview and mental status examination performed by a psychiatrist during outpatient visits as a part of a routine care. The inclusion criteria for patients were as follows: (a) diagnosis of schizophrenia, BD, or MDD in accordance with the criteria of the DSM-5, (b) age ≥ 18 years, (c) patients who were not in the acute phase of the disease, (d) Patients who have lived with their parents for the first 16 years of their life (e) patients whose both of their parents have no history of serious mental illnesses, and (f) patients without any known systemic or neurological diseases that may confound cognitive performance. Illiterate patients, psychiatric hospitalization within the past 6 months, patients with intellectual disabilities (diagnosed by DSM-5) and those who refused to give informed consent or to answer the questionnaire were excluded from the study.

We used the quota sampling method for each disease. Based on the score of parental bonding tool in patients with schizophrenia and bipolar disorder in a study by Gomez et al. [16], using the sample size formula (N = z1-α/2 2 S2/d2 (and assuming that α = 0.05 and d = 0.3S, we specified the sample size of different disorders as follows; at least 43 patients with schizophrenia and a minimum of 43 patients with bipolar. According to the parental bonding scores of depressed patients in a study by Bahreini et al. [19], the sample size for patients with depression was estimated to be at least 44. In performing the pairwise comparison between the parental bonding styles of patients with schizophrenia, bipolar disorder, and depression, the sample size obtained through sample size formula for pairwise comparison, (_*N* = 2(z1-α/2 + z1-β_) _2 S2/d2_, S^2^ = (s_1_^2^ + s_2_^2^) /2, d^2^ = (μ_1_-μ_2_)^2^), was less than that calculated for each group when α = 0.05 and study power = 80%; therefore, the minimum sample size was assumed to be equal to the minimum sample size of each group.

### Data collection

Data were collected from September 2018 to December 2018. To this end, we visited three public psychiatric clinics and a private psychiatric center in Bushehr and explained the research purpose to patients after making sure of the inclusion criteria. Afterwards, the questionnaires were given to eligible patients who had provided the written informed consent. Each patient was asked to complete the questionnaires within almost 10 to 12 min. The measure was completed for both mothers and fathers separately.

For data collection, we used Parker’s parental bonding instrument (PBI). This 25-item instrument is applicable to adolescents who are 16 years or older [22]. The measure is ‘retrospective’, meaning that adults (over 16 years) complete the measure for how they remember their parents during their first 16 years. Of the 25 items, 12 belong to “care” (score range: 0 to 36) and 13 belong to “control” (overprotection) (score range: 0 to 39). Thirteen items were directly scored while 12 items were inversely scored. Direct scoring was conducted in a way that “very like” received a score of 3, “moderately like” received score 2, “moderately unlike” received score 1, and “very unlike” received score 0. In this questionnaire, care items 1, 5, 6, 11, 12, 17 and control items 8, 9, 10, 13, 19, 20, and 23 were directly scored. Care items 2, 4, 14, 16, 18, 24 and control items 3, 5, 7, 15, 21, 22, and 25 were indirectly scored. For mothers, the cut-off scores were 27 and 13.5 for care and control, respectively; for fathers, these scores were 24 and 12.5. In addition to generating care and protection scores for each scale, parents were effectively assigned based on the individual’s responses to one of four quadrants: optimal parenting (low control, high care), affectionless control (low care, high control), affectionate constraint (high care, high control), and neglectful parenting (low care, low control) [13]. The instrument’s validity has been confirmed in various studies and its content validity index was 0.81. Also, its reliability was reported to be suitable for mothers and fathers with Cronbach’s alpha coefficient of 0.79 to 0.88 [23].

### Ethical considerations

This study was approved by the Ethics Committee of the Deputy of Research and Information Technology at Bushehr University of Medical Sciences (IR.BPUMS.REC.1396.40). All procedures performed in study involving human participants were in accordance with the ethical standards of the institutional and/or national research committee and with the 1964 Helsinki declaration and its later amendments or comparable ethical standards. All participants and parents/legal guardians gave their written informed consent after having been enlightened the details of the procedure. Confidentiality of information and anonymity were among the other issues that were emphasized.

### Data analysis

The data were analyzed using SPSS 22 (SPSS, Chicago, IL, USA). Mean, standard deviation, percentages, and frequency were used for data description. Normality of variables was checked using the Shapiro-Wilk test, which showed that they did not follow normal distribution. For inferential analysis, we employed the Chi-square and Kruskal-Wallis tests at a significant level of 0.05.

## Results

We assessed 147 patients based on inclusion and exclusion criteria out of which 130 subjects were eligible and therefore provided consent. All the participants answered all the questions and none chose to withdraw from the study. This study included44 depressed patients with a mean age of 31.09 ± 7.7 years, 43 schizophrenic patients with a mean age of 31.56 ± 5.77 years, and 43 bipolar patients with a mean age of 30.51 ± 7.74 years. Most participants were male (*n* = 73, 56.15%), single (*n* = 76, 58.46%) and had a high school education (*n* = 57, 43.84%) and a history of hospitalization (*n* = 72, 55.38%) (Table 1).

**Table 1:** Demographic characteristics of participants

Demographic characteristics		depression	bipolar	schizophrenia	*p*-value
gender	female	21 (47.7)	20 (46.5)	16 (37.2)	0.559
	male	23 (52.3)	23 (53.5)	27 (62.8)	
Marital status	married	13 (29.5)	15 (34.9)	9 (20.9)	0.082
	divorce	1 (2.3)	7 (16.3)	9 (20.9)	
	single	30 (68.2)	21 (48.8)	25 (58.2)	
Patient Education status	Elementary school	0 (0)	0 (0)	2 (4.7)	0.001
	Under diploma	8 (18.2)	14 (32.5)	27 (62.7)	
	diploma	21 (47.7)	22 (51.2)	14 (32.6)	
	academic	15 (34.1)	7 (16.3)	0 (0)	
Father education status	literate	41 (93.2)	39 (90.7)	35 (81.4)	0.068
	illiterate	2 (6.8)	4 (9.3)	8 (18.6)	
Mother education status	literate	38 (86.4)	32 (74.4)	26 (60.5)	0.023
	illiterate	6 (13.6)	11 (25.6)	17 (39.5)	
Father job	employed	40 (90.9)	38 (88.4)	40 (93)	0.757
	unemployed	4 (9.1)	5 (11.6)	3 (7)	
Mother job	employed	12 (27.3)	15 (34.9)	8 (18.6)	0.235
	unemployed	32 (72.7)	28 (65.1)	35 (81.4)	
History of admission	yes	8 (18.2)	25 (58.1)	39 (90.7)	0.001
	no	36 (81.8)	18 (41.9)	4 (9.3)	

**Fig. 1:**
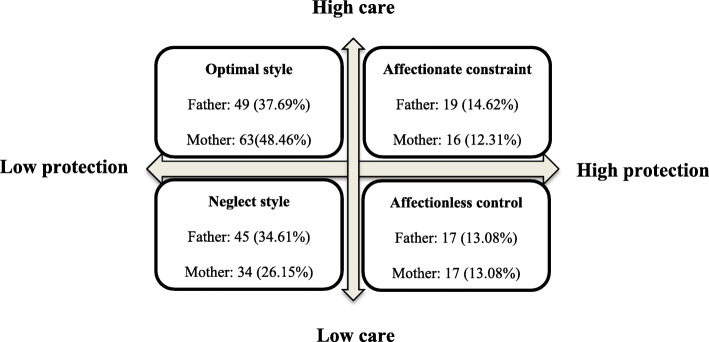
Overall frequency of parental bonding styles in psychiatric patients

Based on the findings, the hypothesis 1 was not supported*.* Accordingly, in all three groups, mean of paternal and maternal care was less than the cut-off point (low care), but there was no significant difference among the patients. However, the means of paternal and maternal control of patients were significantly different (*p* = 0.001 and *p* = 0.024, respectively); as a result, the control scores of both parents of bipolar patients were higher than the depressed and schizophrenic patients (Table 2). These finding supported hypothesis 2. Moreover, in general, 81 patients (62.30%) experienced non optimal paternal styles, and 67 patients (51.54%) experienced non optimal maternal styles. “Neglectful parenting” was also recognized in both fathers and mothers as the most inefficient parenting style (Fig. 1).

**Table 2:** Mean and standard deviation of fundamental parental bonding styles in psychiatric patients

Fundamental parental bonding styles		Depression (M ± SD)	Bipolar (M ± SD)	Schizophrenia (M ± SD)	*p*-value
Mother	care	23.95 ± 5.72	23.28 ± 6.47	25.23 ± 6.90	0.219
	control	16.18 ± 5.67	19.95 ± 7.97	14.14 ± 6.51	0.001
Father	care	23.68 ± 5.02	22.60 ± 7.30	24.58 ± 6.41	0.447
	control	16.77 ± 5.99	19.28 ± 8.75	14.74 ± 6.81	0.024

The comparison of paternal bonding styles among schizophrenic, depressed, and bipolar patients revealed that the most frequent paternal bonding styles were “optimal parenting” followed by “neglectful parenting” with a slight difference among the three groups. The paternal bonding styles did not show any significant difference between the fathers of the three groups (*p* = 0.848) (Table 3). Meanwhile, mothers of patients with schizophrenia (60.5%) used non optimal parental styles more than mothers of depressed and bipolar patients. These results revealed a significant difference between the patients’ maternal bonding styles (*p* = 0.007) (Table 3) and supported hypothesis 3. Statistical analysis indicated no association between the demographics’ of participants and their parental bonding styles.

**Table 3:** Frequency of parental bonding styles in psychiatric patients

Parental bonding styles		DepressionFrequency (%)	BipolarFrequency (%)	SchizophreniaFrequency (%)	${\boldsymbol{x}}_{\left(\mathbf{6}\right)}^{\mathbf{2}}$	*P*-value
Mother	Affectionate constrains	4 (9.1)	1 (2.3)	11 (25.6)	17.66	0.007
	Affectionless control	5 (11.4)	4 (9.3)	8 (18.6)		
	Neglect style	10 (22.7)	17 (39.5)	7 (16.3)		
	Optimal style	25 (56.8)	21 (48.9)	17 (39.5)		
Father	Affectionate constrains	5 (11.4)	5 (11.6)	9 (20.9)	2.67	0.848
	Affectionless control	6 (13.6)	7 (16.3)	4 (9.3)		
	Neglect style	16 (36.4)	15 (34.9)	14 (32.6)		
	Optimal style	17 (38.6)	16 (37.2)	16 (37.2)		

## Discussion

The present study aimed to determine and compare parental bonding styles in the parents of patients with schizophrenia and mood disorders including depression and bipolar disorders. The results showed that the mean parental care was not significantly different among the patients; on the other hand, their mean parental control was significantly different, and the paternal and maternal control scores were higher in bipolar patients compared with schizophrenic patients. In other words, parents of bipolar patients are more protective and controlling. This result is consistent with the studies in European and Latin American countries despite the difference in the cultural and social characteristics [12, 16]. In another study carried out in Japan, subjects who experienced “paternal affectionless control” displayed less ability to adapt with anxiety and stress, leading to mental disorders in these individuals [24]. The attachment theory of Bowlby underscores that inefficient parental style such as excessive control can make children more prone to mental disorders [11].

In addition, the results of the present study indicated no significant differences among patients with schizophrenia, depression, and bipolar disorder in terms of paternal bonding styles. However, most of the patients described their paternal styles as inefficient. Among the inefficient styles, the most prevalent one was “neglectful parenting”, observed in almost one third of the three groups. In this style of parenting, characterized by low protection and care, the father does not have enough control over the children’s behavior, nor does he provide enough care. There is compelling scientific evidence which introduces childhood neglect as a risk factor for mental disorders in adulthood [25]. In a study in Italy, a strong relationship was observed between parents’ emotional neglect and children’s mental disorders [26]. Emotional neglect usually stems from a parent’s failure or refusal to satisfy their children’s psychological needs. Rejection of the children, refusal to interact with them and failure to express feelings are but examples of such behaviors [27]. As a warm and positive parent-child relationship can strengthen children’s self-regulatory abilities [28] and create a sense of security in them [29], a cold and emotionally neglected relationship can preclude positive experiences in children.

The present study also showed a significant difference among the patients in terms of maternal bonding styles; in this regard, the most inefficient style was “affectionate constraint” (high protection, high care) in schizophrenic patients. Some researchers hold that inefficient parental bonding styles contribute to mental disorders by impacting children’s cognitive systems and beliefs. They propose that cognitive beliefs and schemas can predict and determine behaviors and emotional responses based on the hypotheses of cognitive models. In some individuals, these behaviors and emotional responses lead to identity crisis, ineffective defense mechanisms, and maladaptive and pathological behaviors [30]. A study on a group of delinquent prisoners assessed the effects of different parental styles on their beliefs and cognitive schemas. According to their results, most of the prisoners’ parents used affectionate constraint. In this style, parents exert excessive control and care over their children. The prisoners also reported that excessive control caused negative cognitive schemas, such as social isolation and dependence. In fact, this study considered a process that explained the effects of parental bonding styles on the children’s mental health [31].

In terms of parental control, the results of the present study are in line with a study that examined parenting styles in people running high risks of psychosis and a study that investigated schizophrenic patients for parenting styles [32, 33]. Another study in Iran emphasized the relationship between the symptoms of psychiatric disorders and affectionless control parenting (high protection, low care) in parents. Their results also emphasized the relationship between maternal low care styles and psychological symptoms while introducing affectionless control style as a strong predictor of the severity and frequency of mental illness symptoms, especially depression [19]. These contradictory results imply the need for more studies in this field.

In the present study, neglectful style (low protection, low care) was the most common inefficient parenting style among the mothers of depressed patients. One study, conducted in Italy, examined a number of adolescents with “alexithymia”; they showed the relationship between “parental bonding styles” and alexithymia in adolescents. In particular, the maternal care style was associated with problems concerning the “explanation and expression of emotions” in adolescents. These problems, which are obvious features of alexithymia, were observed in the maternal neglectful style. The foregoing study also revealed that inefficient paternal styles, especially in the low care style, could be strong underlying causes of alexithymia. Furthermore, the studied adolescents were susceptible to mental disorders, such as depression, schizophrenia, and anxiety [34]. Alexithymia is known as a personality trait which prevents individuals from regulating their emotions and causes them to have problems with identifying, describing, and interpreting their own and others’ emotions. Some studies have introduced alexithymia as an underlying factor for the development of mental disorders [35, 36]. In addition, some parental bonding styles, low care styles in particular, are positively correlated with alexithymia; therefore, can argue that inefficient parental styles, especially those based on low care, can be considered as underlying factors for the development of mental disorders.

According to our findings, parental bonding in schizophrenic patients has interesting characteristics. These patients perceived their parental bonding as paternal low care/ maternal high care and paternal low control/ maternal high control. In a longitudinal study on groups of patients with anxiety, depression, or both, depressed patients reported that their fathers did not provided adequate levels of “care” during the first 16 years of their lives [37]. In other words, the depressed patients suffered from the lack of a warm, loving, and close relationship with their fathers. A study on a large sample size in the United States also examined the association between parental styles and 13 common psychiatric illnesses; they observed a strong relationship between parental low care and psychiatric disorders [38].

### Strength and limitations

To the best of our knowledge, the present study was the first in Iran to investigate three important psychiatric disorders in terms of their relationship with parental bonding. Similar to other studies, the present study had some limitations. Firstly, a major limitation of this study is the lack of healthy controls. Because there is no comparison with healthy controls, it is difficult to discuss the impact of the parental bonding on the diagnoses. However, comparing our data to the ones in literature on the general population [17–19], it should be noted that psychiatric patients are more often affected by ineffective parental bonding styles. Secondly, this study only examined the southern part of Iran with specific cultural and social characteristics which could influence the parental styles and the participants’ perception of them. Furthermore, the samples were taken through convenience and non-random methods, which would probably affect the generalizability of the results.

## Conclusions

The results of the study emphasized that parenting styles could be considered as predictors and predisposing factors for mental disorders. Moreover, all patients received low maternal care and bipolar patients experienced excessive control from both parents. These results shed more light on the important role of parents in developing the children’s mental health by emphasizing childhood and adolescence. Our findings further confirmed that while there are several biological and psychosocial factors involved in the development of mental disorders, the role of parents, especially mothers, should be considered in mental health promotion strategies. Planners and executers of mental health programs should revise family education programs and provide necessary training for families on the importance of parents’ appropriate and optimum care and control for their children.

## Supplementary Information

**Additional file 1.** Parental Bonding Instrument (PBI).

## Data Availability

The datasets used and/or analyzed during the current study are available from the corresponding author on reasonable request.

## Abbreviations

PBI: Parental Bonding Instrument
BD: Bipolar Disorder
MDD: Major Depressive Disorder
